# Anaphylactic response to topical fluorescein 2% eye drops: a case report

**DOI:** 10.1186/1752-1947-4-27

**Published:** 2010-01-29

**Authors:** Humma Shahid, John F Salmon

**Affiliations:** 1 Oxford Eye Hospital, West Wing, Oxford Radcliffe Hospitals NHS Trust, Oxford, OX3 9DU, UK

## Abstract

**Introduction:**

The intravenous use of fluorescein 10% during retinal angiography can cause severe systemic reactions including, on rare occasions, anaphylaxis. Fluorescein 2% eye drops are used extensively for clinical examination and diagnosis, but to the best of our knowledge, they have only been reported as being responsible for a systemic anaphylactic response on two previous occasions.

**Case presentation:**

We report the case of a 51-year-old woman who developed an anaphylactic reaction when she was administered fluorescein sodium 2% eye drops after cataract surgery. This was the second time she had been exposed to fluorescein. She had brittle asthma and a history of anaphylaxis following exposure to a variety of drug and food allergens. She was successfully resuscitated and recovered completely over a period of two days.

**Conclusions:**

Fluorescein 2% drops are universally used in general practice, ophthalmology, optometry, and casualty departments. Our case report reveals the potential for this benign eye drop to cause a life-threatening systemic reaction and emphasises the importance of considering this consequence when administering topical fluorescein 2% to a patient with a history of anaphylaxis to other allergens.

## Introduction

The most common cause of a life-threatening medical emergency in outpatient ophthalmology clinics is the administration of intravenous fluorescein 10% during retinal angiographic investigations. Most of the side effects of intravenous fluorescein are relatively benign, and the most common reactions are nausea (2.9%), vomiting (1.2%), and a combination of flushing, itching or urticaria (0.5%) [1]. The frequency of severe reactions to intravenous fluorescein administration, including laryngeal oedema, bronchospasm, myocardial infarction, cardiac arrest and tonic clonic seizure, is much lower at 0.05% [2]. A recent study noted that there were no serious adverse events or deaths reported from a series of 11,898 fluorescein angiogram studies [3]. The risk of an anaphylactic reaction to fluorescein during fundus fluorescein angiography has an extremely low occurrence, with one death reported from 220,000 investigations [2].

In most circumstances, an allergic reaction to eye drops involves a mild localised ocular allergic reaction which resolves when the drops are discontinued [4]. Reports of anaphylactic reactions following the topical administration of medicines to the eye are rare but potentially fatal. In this report we present the third known case of a systemic anaphylactic reaction following the topical administration of fluorescein sodium 2% [5,6].

## Case presentation

A 51-year-old English woman attended the Oxford Eye Hospital for a postoperative check-up two weeks after uneventful phacoemulsification cataract surgery that was performed under topical anaesthesia. As part of the ophthalmic examination, fluorescein 2% eye drops (Bausch and Lomb Minims^®^) were administered topically into her operated eye in order to check the corneal wound integrity and measure the intraocular pressure. No other eye drops were instilled into her eye during her outpatient visit. Within 30 seconds of topical fluorescein administration, she developed acute dyspnoea, wheezing and tachycardia. Well-versed with the symptoms of the onset of anaphylaxis, she administered 0.6 mg intramuscular adrenaline from her auto-injector (EpiPen^®^). Venous access was obtained and the patient was given intravenous chlorpheniramine and oral prednisolone. Despite a good initial response, she deteriorated acutely within 30 minutes, with tongue swelling, airway obstruction, and subsequent loss of consciousness. Following appropriate resuscitation, she was transferred for further observation to the intensive care unit. She was discharged from our hospital after 48 hours.

Our patient was known to have brittle asthma and a history of allergy to a number of drugs (Appendix 1) and foods (Appendix 2). She suffered 10 episodes of anaphylaxis following inadvertent exposure to these allergens in the preceding year, one of which necessitated admission to an intensive care unit. She was examined in the ophthalmology department on two occasions before the visit described above. At the preoperative assessment prior to cataract surgery, the intraocular pressure was measured using a Tono-pen^® ^applanation tonometer using topical proxymetacaine drops. Cataract surgery was subsequently performed without any complication under topical anaesthesia (with benoxinate, proxymetacaine and amethocaine drops). Before she was discharged from the ophthalmology day surgery unit, the operated eye was examined and fluorescein drops were instilled for the first time. No systemic reaction to fluorescein was observed at this time.

Two months after the anaphylactic reaction to topical fluorescein 2%, our patient returned for cataract surgery on her other eye. No fluorescein was administered at any stage and she had an uneventful postoperative recovery.

## Discussion

Anaphylaxis is defined as a severe, life-threatening, generalised or systemic hypersensitivity reaction affecting two or more organs or systems [7]. During the sensitisation phase of this immediate (type I) hypersensitivity reaction, an allergen triggers the formation of specific Immunoglobulin E molecules, which then bind to high affinity FcεR1 receptors on the surface of tissue mast cells, basophils, and eosinophils. During the challenge phase, exposure to the same allergen results in the cross-linking of the membrane-bound Immunoglobulin E molecules on the "sensitised" cells. This causes degranulation and the release of both the preformed and newly synthesised pharmacologically active mediators of the anaphylactic reaction. The measurement of serum beta-tryptase released from activated mast cells can be detected by radioimmunoassay and serves as a specific marker for anaphylaxis [8].

A true anaphylactic reaction to topical fluorescein 2% is extremely rare. To the best of our knowledge, only two previous reports describe an anaphylactic reaction following the instillation of fluorescein to the eye [5,6]. Topical fluorescein is widely used in the diagnosis of corneal abrasions, ulcers, herpetic eye disease, and corneal wound leaks. It is also used in measuring intraocular pressure and assessing contact lens fit. The conjunctiva contains lymphoid tissue in the stroma and has a rich vascular and lymphatic supply, and can thus be involved in a regional hypersensitivity response to a foreign antigen. Given the widespread use of topical fluorescein, it is not known why the incidence of anaphylaxis to this drug is so rare. Although the conjunctiva acts as a barrier to the penetration of molecules, animal studies have shown that compounds of molecular weight less than 3,496 daltons are required to elicit ocular anaphylaxis when applied topically. With a molecular weight of 376 daltons, sodium fluorescein can easily pass through the conjunctiva [9]. One could speculate that by increasing the molecular size of fluorescein, perhaps through the addition of an inert molecular chain, conjunctival absorption, and therefore the potential of fluorescein to cause an anaphylactic reaction, could be eliminated.

Our patient was known to be hypersensitive to a variety of allergens and had brittle asthma. These factors are known to be associated with increased fatality following an anaphylactic reaction [10]. A strong history of previous anaphylaxis in a patient indicates the need to exercise caution when administering new medications by any route. The first administration of topical fluorescein was on the day of the cataract surgery. No reaction was elicited at this point, and this was probably due to the sensitising dose of fluorescein. The second administration of fluorescein then caused the occurrence of an anaphylactic reaction.

## Conclusions

This case illustrates how the administration of a seemingly benign fluorescein 2% eye drop can have serious consequences in a susceptible patient. The absence of an anaphylactic reaction when topical fluorescein is used for the first time does not preclude a life-threatening reaction from its subsequent administration in the same patient. Given the widespread use of fluorescein eye drops across specialties in primary and secondary care settings, doctors and other ophthalmic practitioners need to be aware of this rare response to topical fluorescein. It is important to take a good medical and drug history for every patient. In particular, a reported history of multiple drug and food allergies is a significant finding, especially if these allergens have triggered anaphylaxis on previous occasions.

## Consent

Written informed consent was obtained from the patient for publication of this case report and any accompanying images. A copy of the written consent is available for review by the Editor-in-Chief of this journal.

## Competing interests

The authors declare that they have no competing interests.

## Authors' contributions

HS conducted the literature review and wrote the manuscript. JFS critically reviewed the manuscript. Both authors read and approved the final manuscript.

## Appendix 1 - Patient's drug allergies

Aminophylline

Cocaine

Lignocaine

Metformin

Ranitidine

All medications containing sodium metabisulphite (E223) including paracetamol

## Appendix 2 - Patient's food allergies

All nuts

Shellfish

Mushrooms (canned or dried)

All lettuce (except Iceberg)

Apples

Pears

Dehydrated vegetables

Watercress

Pre-packed foods containing artificial preservatives

Sausages and processed meat

Fruits with stones (peaches, nectarines)

Food additives E220-E228 and E150

Celery

Pepper

Mustard
